# Risk of Neurodevelopmental Disorders in Offspring of Parents with Major Depressive Disorder: A Birth Cohort Study

**DOI:** 10.1007/s10803-024-06502-3

**Published:** 2024-08-01

**Authors:** Yu-Han Lin, Shih-Jen Tsai, Ya-Mei Bai, Tzeng-Ji Chen, Mu-Hong Chen

**Affiliations:** 1 Department of Psychiatry, Taipei Veterans General Hospital, No. 201, Shih-Pai Road, Sec. 2, Taipei, 11217 Taiwan; 2 Department of Psychiatry, College of Medicine, National Yang Ming Chiao Tung University, Taipei, Taiwan; 3 Department of Family Medicine, Taipei Veterans General Hospital, Taipei, Taiwan; 4 Institute of Hospital and Health Care Administration, National Yang Ming Chiao Tung University, Taipei, Taiwan; 5 Department of Family Medicine, Taipei Veterans General Hospital, Hsinchu Branch, Hsinchu, Taiwan

**Keywords:** Parental depression, Offspring, Developmental delay, Autism, ADHD

## Abstract

Studies have reported inconsistent results regarding associations between parental depression and offspring neurodevelopmental disorders, such as developmental delay and autism spectrum disorder (ASD). In all, 7,593 children who were born between 1996 and 2010 in Taiwan and had at least one parent with major depressive disorder and 75,930 birth-year- and sex-matched children of parents without major depressive disorder were followed from 1996 or time of birth to the end of 2011. Intergroup differences in neurodevelopmental conditions—including ASD, attention-deficit hyperactivity disorder (ADHD), tic disorder, developmental delay, and intellectual disability (ID)—were assessed. Compared with the children in the control group, the children of parents with major depression were more likely [hazard ratio (HR), 95% confidence interval (CI)] to develop ADHD (1.98, 1.80–2.18), ASD (1.52, 1.16–1.94), tic disorder (1.40, 1.08–1.81), developmental delay (1.32, 1.20–1.45), and ID (1.26, 1.02–1.55). Parental depression was associated with offspring neurodevelopmental disorders, specifically ASD, ADHD, developmental delay, ID, and tic disorder. Therefore, clinicians should closely monitor the neurodevelopmental conditions of children of parents with depression.

## Introduction

Major depressive disorder has long been notorious for causing patients to experience profound impairments in various aspects, including learning, occupational functioning, and social interactions (Malhi et al., 2018; Kupfer et al., 2012). Furthermore, there appears to be a connection between individuals suffering from depression and the risk of neurodevelopmental disorders in their children, although the causal relationship between them remains unclear (Malhi et al., 2018; Kupfer et al., 2012). The evidence indicates that the offspring of individuals with major depression are at risk of various neurodevelopmental disorders, including attention deficit hyperactivity disorder (ADHD) autism spectrum disorder (ASD), and various forms of developmental delays (e.g., intellectual disability [ID]) (Gul et al., 2020; Morgan et al., 2012; Ayano et al., 2019; Vizzini et al., 2019).

In a cross-sectional study that employed the brief Infant–Toddler Social and Emotional Assessment Scale and Ankara Developmental Screening Inventory to examine 79 infant–mother dyads, maternal depression was revealed to be associated with various types of developmental delays in infants, including cognitive language, fine motor, gross motor, and social delays (Gul et al., 2020). A cohort study of 1,255 children of mothers with major depression and 3,129 children of mothers without major depression reported that the children in the maternal depression group had a significantly higher risk of ID [odds ratio (OR), 2.9] (Morgan et al., 2012). Studies have also suggested that maternal depression is associated with risks of ASD and ADHD in offspring (Ayano et al., 2019; Vizzini et al., 2019). A meta-analysis of two cohort studies and seven case–control studies reported that children whose parents had a major affective disorder (e.g., major depression) had a higher risk of ASD relative to those whose parents had no psychiatric disorders (Ayano et al., 2019). The NINFEA birth cohort study, which involved 3,634 mother–singleton dyads, reported that maternal lifetime depression was associated with ADHD symptoms in 4-year-old offspring (Vizzini et al., 2019). In addition, Shlomo et al. indicated that maternal depression increased the risk of tic disorder in children (Ben-Shlomo et al., 2016). However, the aforementioned studies mostly involved participants of European origin, which is a factor that limits the generalizability of the associations between parental depression and offspring neurodevelopmental disorders to Asian populations.

In our study, we used data from Taiwan National Health Insurance Research Database (NHIRD) to establish a large sample and adopted a longitudinal follow-up study design to assess the associations between parental depression and offspring neurodevelopmental conditions, including ASD, ADHD, tic disorder, and developmental delay. We hypothesized that the offspring of parents with major depression are more likely to develop ASD, ADHD, tic disorder, and various types of neurodevelopmental delay relative to those of parents without major depression.

## Methods

### Data Source

Taiwan’s National Health Insurance, a mandatory universal health insurance program, was implemented in 1995 and offers comprehensive medical care coverage to all Taiwanese residents. The Taiwan NHIRD, which comprises healthcare data from > 99.7% of Taiwan’s population, were audited and released by the Taiwan National Health Research Institute for scientific studies. The insurance claim information of the subjects is anonymous to maintain privacy. Comprehensive information on insured subjects is included in the database, such as demographic data, clinical visit dates, disease diagnoses, and prescriptions. The diagnostic codes used were based on the International Classification of Diseases, 9th Revision, Clinical Modification (ICD-9-CM). The National Health Insurance Research Database has been used extensively in many Taiwanese epidemiologic studies (Chen et al., 2016, 2018a, b; Zhang et al., 2021). This study protocol was reviewed and accepted by the Institutional Review Board of our Hospital.

### Inclusion Criteria for the Offspring of Parents with Major Depression

Individuals who were born between 1996 and 2010 and had any parent with major depression (ICD-9-CM codes: 296.2 and 296.3) given by board-certified psychiatrists were selected as the study group in current study. The present study excluded those who had any parents with other major psychiatric disorders, including schizophrenia and bipolar disorder. The age-, sex-, birth time-, residence- and family income-matched (1:10) control cohort was randomly identified after eliminating the study cases and those who had any parent with severe mental disorders, including schizophrenia, bipolar disorder, and major depressive disorder. We also assessed the timing (prenatal vs. postnatal) of parental depression. We classified individuals exposed to any parent with major depression prior to their births as the prenatal exposure group. Individuals in the study group who were not exposed to prenatal depression were classified as the postnatal exposure group. Those in the study group who were exposed to parental depression at both prenatal and postnatal periods were also classified as the prenatal exposure group. The study cohort and control cohort were followed from 1996 or the birth time to the end of 2011 for the investigation of occurrence of neurodevelopmental disorders, including ASD, ADHD, tic disorder, and developmental delay conditions. Developmental delay conditions, including any developmental delay, developmental speech or language disorder, developmental coordination disorder, and ID, were diagnosed at least twice by board-certified psychiatrists, pediatricians, and rehabilitation medicine physicians. ADHD and ASD were diagnosed at least twice by board-certified psychiatrists, and tic disorder was diagnosed at least twice by board-certified psychiatrists, pediatricians, and neurologists. Comorbid perinatal conditions, including maternal causes of perinatal morbidity, preterm or low birth weight, respiratory distress, birth trauma, and neonatal jaundice, were identified as the confounding factors. Level of urbanization (level 1 to level 5; level 1: most urbanized region; level 5: least urbanized region) was also assessed for our study (Liu et al., 2006). Additionally, Charlson Comorbidity Index (CCI) and all-cause clinical visits were provided for the study and the matched-control cohorts. CCI consisting of 22 physical conditions was also assessed to determine the systemic health conditions of all enrolled subjects (Charlson et al., 1987). This study was approved by the Institutional Review Board of Taipei Veterans General Hospital.

### Statistical Analysis

For between-group comparisons, the F test was used for continuous variables and Pearson’s X^2^ test for nominal variables, where appropriate. Cox regression analyses with adjustment of demographic data (age, sex, residence, and income), comorbid perinatal conditions (maternal causes of perinatal morbidity, preterm or low birth weight, respiratory distress, birth trauma, and neonatal jaundice), CCI scores, and all-cause clinical visit were performed to examine the subsequent likelihoods of neurodevelopmental disorders (ASD, ADHD, tic disorder, any developmental delay, developmental speech or language disorder, developmental coordination disorder, and ID) in the offspring of parents with major depression compared with those having no parents with major mental disorders. Furthermore, we clarified associations between the timing of parental depression and the risks of offspring neurodevelopmental conditions. A 2-tailed *P*-value of less than 0.05 was considered statistically significant. All data processing and statistical analyses were performed with Statistical Package for Social Science (SPSS) version 17 software (SPSS Inc.) and Statistical Analysis Software (SAS) version 9.1 (SAS Institute, Cary, NC).

## Results

In all, 7593 children who had any parent with major depression and 75,930 age- and sex-matched children who had no parents with any severe mental disorder were included in our study. Children of parents with major depression had a higher incidence of developing any neurodevelopmental condition, including ASD (0.9% vs. 0.5%, *p* < 0.001), ADHD (6.5% vs. 3.2%, *p* < 0.001), tic disorder (0.9% vs. 0.6%, *p* = 0.003), and any developmental delay (6.2% vs. 4.4%, *p* < 0.001), compared with those of control parents (Table 1). In addition, the comorbid perinatal conditions, such as preterm or low birth weight (3.3% vs. 2.6%, *p* < 0.001), respiratory distress (3.6% vs. 3.0%, *p* = 0.003), and neonatal jaundice (8.5% vs. 7.5%, *p* = 0.001), were higher in the study group than in the control group (Table 1).

**Table 1:** Demographic characteristics and incidence of neurodevelopmental disorders between the offspring of parents with or without major depressive disorder

	Offspring of parents with major depressive disorder (n = 7593)	Offspring of parents without major depressive disorder (n = 75,930)	p-value
Birth year (n, %)			0.773
1996 ~ 1999	4157 (54.7)	41,303 (54.4)	
2000 ~ 2005	2891 (38.1)	29,228 (38.5)	
2006 ~ 2010	545 (7.2)	5399 (7.1)	
Male (n, %)	4024 (53.0)	40,240 (53.0)	1.000
Parental major depressive disorder (n, %)			
Fathers	2745 (36.2)		
Mothers	4996 (65.8)		
Timing of parental major depressive disorder (n, %)			
Prenatal (prior to births)	3792 (49.9)		
Postnatal (after births)	3801 (50.1)		
Incidence of neurodevelopmental disorders (n, %)			
ASD	65 (0.9)	381 (0.5)	< 0.001
Age at diagnosis (years, SD)	6.06 (3.43)	5.74 (3.15)	0.453
ADHD	497 (6.5)	2446 (3.2)	< 0.001
Age at diagnosis (years, SD)	7.47 (2.27)	7.58 (2.34)	0.357
Tic disorder	67 (0.9)	452 (0.6)	0.003
Age at diagnosis (years, SD)	8.13 (2.25)	7.67 (2.48)	0.149
Any developmental delay	474 (6.2)	3313 (4.4)	< 0.001
Age at diagnosis (years, SD)	4.28 (2.51)	4.25 (2.31)	0.766
Developmental speech or language disorder	279 (3.7)	2176 (2.9)	< 0.001
Age at diagnosis (years, SD)	4.11 (2.15)	4.11 (1.89)	0.983
Developmental coordination disorder	107 (1.4)	550 (0.7)	< 0.001
Age at diagnosis (years, SD)	5.67 (2.29)	5.23 (2.04)	0.048
Intellectual disability	103 (1.4)	734 (1.0)	0.002
Age at diagnosis (years, SD)	6.65 (2.64)	6.17 (2.85)	0.104
Comorbid perinatal conditions (n, %)			
Maternal causes of perinatal morbidity	87 (1.1)	802 (1.1)	0.449
Preterm or low birth weight	250 (3.3)	1975 (2.6)	< 0.001
Respiratory distress	277 (3.6)	2296 (3.0)	0.003
Birth trauma	21 (0.3)	264 (0.3)	0.352
Neonatal jaundice	647 (8.5)	5658 (7.5)	0.001
CCI scores (SD)	0.48 (0.64)	0.41 (0.59)	< 0.001
Level of urbanization (n, %)			1.000
1 (most urbanized)	1288 (17.0)	12,880 (17.0)	
2	2421 (31.9)	24,210 (31.9)	
3	840 (11.1)	8400 (11.1)	
4	758 (10.0)	7580 (10.0)	
5 (most rural)	2286 (30.0)	22,860 (30.0)	
Income-related insured amount (n, %)			1.000
≤ 19,100 NTD/month	1723 (22.7)	17,230 (22.7)	
19,001 ~ 42,000 NTD/month	2980 (39.2)	29,800 (39.2)	
> 42,000 NTD/month	2890 (38.1)	28,900 (38.1)	
All-cause clinical visits (times per year, SD)	6.65 (5.34)	6.00 (5.13)	< 0.001

SD: standard deviation; NTD: new Taiwan dollars; ASD: autism spectrum disorder; ADHD: attention deficit hyperactivity disorder; CCI: Charlson Comorbidity Index

**Fig. 1:**
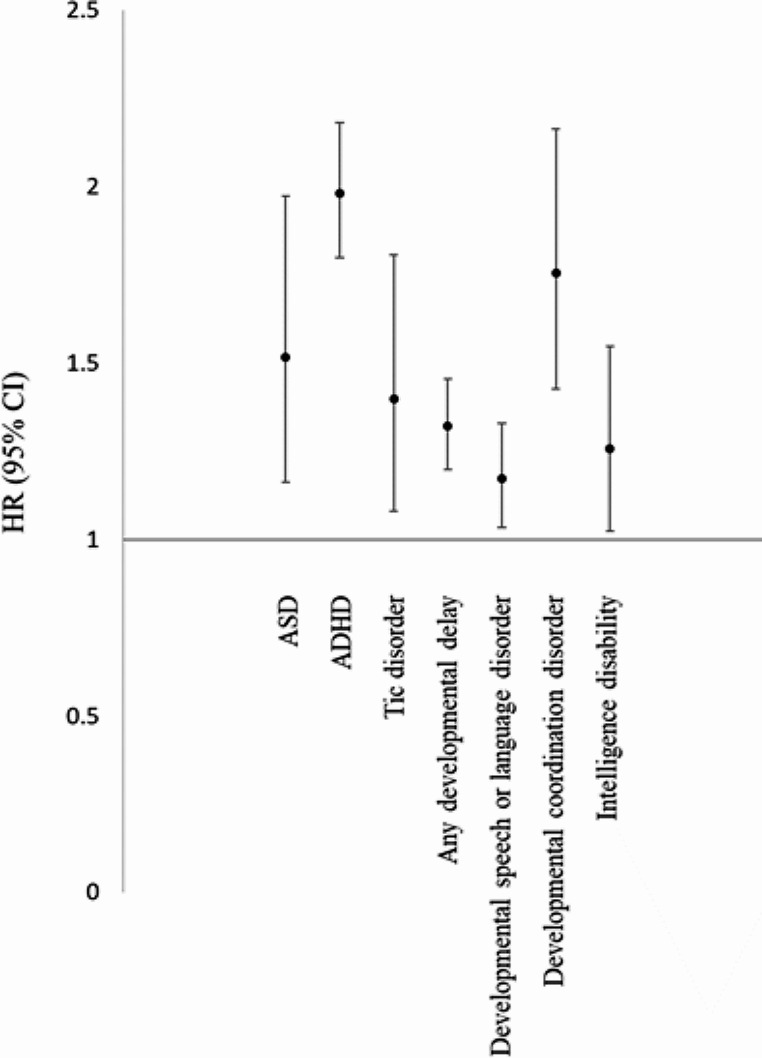
HRs of subsequent neurodevelopmental disorders between the offspring of parents with or without major depressive disorder. HR: hazard ratio; CI: confidence interval; ASD: autism spectrum disorder; ADHD: attention deficit hyperactivity disorder

Cox regression analyses with adjustment of demographic data, comorbid perinatal conditions, CCI scores, and all-cause clinical visit reported that parental depression was associated with increased offspring risks of ASD (hazard ratio [HR]: 1.52, 95% confidence interval [CI]: 1.16–1.94), ADHD (1.98, 1.80–2.18), and tic disorder (1.40, 1.08–1.81) (Table 2; Fig. 1). Furthermore, parental depression was associated with any developmental delay (1.32, 1.20–1.45), developmental speech or language disorder (1.17, 1.03–1.33), developmental coordination disorder (1.76, 1.43–2.16), and ID (1.26, 1.02–1.55) (Table 3; Fig. 1). Specifically, we found that offspring who were exposed to prenatal parental depression were more likely to have diagnoses of ASD (HR: 1.76, 95% CI: 1.23–2.53), ADHD (1.95, 1.70–2.25), tic disorder (1.52, 1.05–2.20), any developmental delay (1.40, 1.23–1.49), developmental speech or language disorder (1.29, 1.09–1.51), and developmental coordination disorder (1.73, 1.31–2.30) than were the control group (Tables 2 and 3). However, postnatal parental depression was only associated with offspring ADHD (HR: 2.00, 95% CI: 1.77–2.27), any developmental delay (1.24, 1.07–1.42), and developmental coordination disorder (1.78, 1.34–2.37) (Tables 2 and 3).

**Table 2:** Risks of subsequent ASD, ADHD, and tic disorder between the offspring of parents with or without major depressive disorder*

	ASD	ADHD	Tic disorder
Offspring of parents without severe mental disorders (n, %)	381 (0.5)	2446 (3.2)	452 (0.6)
Offspring of parents with major depressive disorder (n, %)	65 (0.9)	497 (6.5)	67 (0.9)
HR (95% CI)	**1.52 (1.16–1.94)**	**1.98 (1.80–2.18)**	**1.40 (1.08–1.81)**
Offspring of parents with major depressive disorder (n, %)			
Prenatally exposed	33 (0.9)	210 (5.5)	30 (0.8)
HR (95% CI)	**1.76 (1.23–2.53)**	**1.95 (1.70–2.25)**	**1.52 (1.05–2.20)**
Postnatally exposed	32 (0.8)	287 (7.6)	37 (1.0)
HR (95% CI)	1.32 (0.92–1.90)	**2.00 (1.77–2.27)**	1.31 (0.94–1.84)
Comorbid perinatal conditions (HR, 95% CI)			
Maternal causes of perinatal morbidity	0.43 (0.13–1.37)	1.30 (0.98–1.71)	0.57 (0.21–1.54)
Preterm or low birth weight	1.27 (0.73–2.22)	**1.35 (1.09–1.66)**	1.08 (0.62–1.90)
Respiratory distress	0.87 (0.51–1.47)	0.99 (0.81–1.21)	1.15 (0.70–1.89)
Birth trauma	1.15 (0.28–4.65)	1.19 (0.71–1.98)	0.51 (0.07–3.67)
Neonatal jaundice	**1.39 (1.03–1.88)**	**1.21 (1.07–1.37)**	1.05 (0.77–1.45)

HR: hazard ratio; CI: confidence interval; ASD: autism spectrum disorder; ADHD: attention deficit hyperactivity disorder

*: adjusting for demographic data, comorbid perinatal conditions, CCI scores, and all-cause clinical visits

**Bold** indicates statistical significance

**Table 3:** Risks of subsequent developmental disorders between the offspring of parents with or without major depressive disorder*

	Any developmental delay	Developmental speech or language disorder	Developmental coordination disorder	Intellectual disability
Offspring of parents without severe mental disorders (n, %)	3313 (4.4)	2176 (2.9)	550 (0.7)	734 (1.0)
Offspring of parents with major depressive disorder (n, %)	474 (6.2)	279 (3.7)	107 (1.4)	103 (1.4)
HR (95% CI)	**1.32 (1.20–1.45)**	**1.17 (1.03–1.33)**	**1.76 (1.43–2.16)**	**1.26 (1.02–1.55)**
Offspring of parents with major depressive disorder (n, %)				
Prenatally exposed	261 (6.9)	159 (4.2)	54 (1.4)	44 (1.2)
HR (95% CI)	**1.40 (1.23–1.49)**	**1.29 (1.09–1.51)**	**1.73 (1.31–2.30)**	1.33 (0.98–1.80)
Postnatally exposed	213 (5.6)	120 (3.2)	53 (1.4)	59 (1.6)
HR (95% CI)	**1.24 (1.07–1.42)**	1.05 (0.87–1.26)	**1.78 (1.34–2.37)**	1.21 (0.93–1.58)
Comorbid perinatal conditions (HR, 95% CI)				
Maternal causes of perinatal morbidity	1.03 (0.81–1.31)	1.06 (0.77–1.46)	1.12 (0.64–1.95)	1.22 (0.81–1.84)
Preterm or low birth weight	**2.27 (1.94–2.65)**	**1.75 (1.42–2.16)**	**2.38 (1.65–3.43)**	**2.18 (1.61–2.95)**
Respiratory distress	**1.43 (1.23–1.67)**	1.19 (0.97–1.46)	1.10 (0.76–1.61)	**1.63 (1.21–2.19)**
Birth trauma	**1.74 (1.20–2.51)**	1.54 (0.95–2.49)	**2.73 (1.34–5.53)**	**2.71 (1.52–4.84)**
Neonatal jaundice	**1.15 (1.03–1.27)**	**1.16 (1.01–1.32)**	1.04 (0.80–1.31)	**1.26 (1.02–1.57)**

HR: hazard ratio; CI: confidence interval

*: adjusting for demographic data, comorbid perinatal conditions, CCI scores, and all-cause clinical visits

**Bold** indicates statistical significance

Finally, Tables 2 and 3 demonstrated associations between comorbid perinatal conditions and offspring neurodevelopmental conditions, such as preterm or low birth weight and ADHD (HR: 1.35, 95% CI: 1.09–1.66), various developmental delays (HRs between 1.75 and 2.38), and ID (2.18, 1.61–2.95); neonatal jaundice and ASD (1.39, 1.03–1.88), as well as ADHD (1.21, 1.07–1.37).

## Discussion

Our findings support our study hypothesis that parental depression is associated with an increased risk of developmental disorders in offspring, including developmental speech or language disorder, developmental coordination disorder, ASD, ADHD, ID, and tic disorder. Of those disorders, ADHD had the strongest association with parental depression (HR = 1.98), whereas developmental speech or language disorder had the weakest association with this condition (HR = 1.17).

Numerous studies have investigated the association of parental depression, especially maternal depression, with offspring developmental delay. In a study involving 1,053 mother–infant (aged 6–18-month-old) dyads, Vameghi et al. reported significant associations of maternal depression with overall offspring developmental delay and developmental delays in gross-motor and problem-solving skills (Vameghi et al., 2015). Through the Preschool Language Scale-5, Yoldaş et al. discovered substantial language delay among 1–3-year-old children of mothers with depression (Celen Yoldas & Ozmert, 2021). They also reported that those with language delay had less interaction time and engaged in fewer coviewing activities with their parents (Celen Yoldas & Ozmert, 2021). Morgan et al. discovered that children of mothers with severe mental disorders—including schizophrenia, bipolar disorder, and major depression—were all at a significantly increased risk of ID (Morgan et al., 2012).

An increasing body of evidence is suggesting that parental depression is associated with ASD and ADHD in offspring. Previous family history studies of autism have consistently revealed a subgroup people (i.e., autistic who can function with limited assistance) with a high prevalence of major mood disorder in family members, suggesting the two entities are related clinically and genetically (DeLong, 2004; Cohen & Tsiouris, 2006). Ayano et al. reported an increased risk of ASD in children of mothers with a history of depressive disorder (relative risk, 1.62; 95% CI, 1.32–1.99) but did not identify an association between paternal depression and offspring ASD risk (Ayano et al., 2019). Chien et al. reported that offspring ASD was associated with paternal (OR, 1.17; 95% CI, 1.07–1.27) and maternal (OR, 1.45; 95% CI, 1.35–1.55) depression (Chien et al., 2023). Furthermore, the Norwegian Mother and Child Cohort Study, which examined 17,070 extended-family units, revealed that maternal depression influences the ADHD symptoms of offspring, suggesting that shared genetic and environmental factors contribute to this association (Eilertsen et al., 2021). Nidey et al. reported that children born to mothers with depressive disorder had an increased risk (HR, 3.16; 95% CI, 2.35–4.23) of ADHD during a 5-year follow-up (Nidey et al., 2021). A longitudinal study of 2,620 Australian children and their fathers revealed that paternal depression was associated with poor child behavioral outcomes, including hyperactivity (OR, 1.95; 95% CI, 1.77–2.14), emotional dysregulation (OR, 1.39; 95% CI, 1.27–1.53), and conduct problems (OR, 1.22; 95% CI, 1.11–1.34) (Fletcher et al., 2011). Our findings also support the associations of parental depression with offspring ASD and ADHD. Furthermore, we found associations between prenatal parental depression and offspring autism, as well as offspring ADHD, whereas we only noticed an association between postnatal depression and offspring ADHD.

Notably, few studies have investigated the risk of tic disorder in the offspring of parents with depression (Ben-Shlomo et al., 2016). As was discussed earlier, the Avon Longitudinal Study of Parents and Children reported that parental depressive disorder was associated with the development of tic disorder in children at the age of 13 years (Ben-Shlomo et al., 2016). A nested case–control study of 1,120 children with tic disorder and 4,299 children without tic disorder revealed that maternal affective disorders (OR, 2.3; 95% CI, 1.8–2.9) were associated with the development of tic disorder in these children (Leivonen et al., 2017). Abdulkadir et al. indicated that a polygenic risk score for tic disorder in children was associated with maternal depression (Abdulkadir et al., 2023). The findings of the aforementioned studies align with our finding that parental depression is positively associated with offspring tic disorder. In addition, our study revealed the timing of parental depression with the different risks of offspring neurodevelopmental conditions and tic disorder. Specifically, prenatal parental depression was associated with offspring tic disorder, any developmental delay, developmental speech or language disorder, and developmental coordination disorder. However, postnatal depression was only associated with any developmental delay and developmental coordination disorder.

As expected, prenatal parental depression had a broader impact on the offspring’s neurodevelopment compared with postnatal parental depression. In particular, our findings revealed associations between prenatal depression and neurodevelopmental disorders (both autism and ADHD), as well as various neurodevelopmental delays, in their offspring, whereas postnatal parental depression was only associated with offspring ADHD and developmental coordination disorder. The additive effects of transgenerational genetic and transplacental (i.e., maternal immune activation) risks may explain associations between prenatal parental depression and offspring neurodevelopmental problems (Ayano et al., 2019; Vizzini et al., 2019; Wang et al., 2022; Hall et al., 2023; Solek et al., 2018). Postnatal parental depression-related neurodevelopmental risk in the offspring may highlight a genetic coaggregation between parental depression and offspring autism and ADHD, as well as developmental coordination disorder (Wang et al., 2022; Chen et al., 2021).

Finally, the present study demonstrated the harmful effects of several perinatal conditions, including preterm or low birth weight, neonatal jaundice, newborn respiratory distress, and birth trauma, on the offspring neurodevelopmental problems (Jenabi et al., 2020; Maimburg et al., 2010). A meta-analysis study showed a considerable correlation between neonatal jaundice and autism among children, with a pooled estimate of relative risk of 1.39 (95% CI: 1.05–1.74) (Jenabi et al., 2020). Maimburg et al. discovered that neonatal jaundice was associated with subsequent developmental disorders of speech and language, as well as developmental disorders of motor function (Maimburg et al., 2010). Furthermore, preterm birth and associated symptoms, such as respiratory distress, are convincing risk factors for infant and long-term neurodevelopment (Hee Chung et al., 2020; Song, 2023; Broring et al., 2018; Crump et al., 2021; Lindstrom et al., 2011). Bröring et al. revealed that school-age children born very preterm exhibited higher levels of ADHD and autism symptoms, particularly inattention and difficulties in socialization and communication (Broring et al., 2018). A Swedish national cohort study of 1,180,616 children revealed that preterm and early term birth increased the ADHD risk by degree of immaturity at birth, from OR = 2.1 (95% CI: 1.4–2.7) for 23 to 28 weeks’ gestation, to 1.6 (1.4–1.7) for 29 to 32 weeks’, and 1.4 (1.2–1.7) for 33 to 34 weeks’ gestation compared with infants born at 39 to 41 weeks’ gestation (Lindstrom et al., 2011).

Our study has several strengths. First, we enrolled a large cohort of more than 7,000 parent–child dyads and a matched control cohort comprising more than 70,000 individuals, all of whom were followed prospectively for up to 15 years. Second, in our sample, all cases of major depression were diagnosed by a board-certified psychiatrist, and all developmental disorders were diagnosed at least twice by a board-certified specialist, thereby ensuring the diagnostic validity of our study. However, several study limitations should also be addressed. First, our retrospective cohort design did not allow us to identify causal relationships or clarify the underlying mechanisms between parental depression and the risk of offspring developmental disorders, even though significant associations between them were identified.

Second, the NHIRD only included individuals, such as parents and their offspring, who sought medical and mental consultations. Therefore, our database study classified parents with a mental health problem who did not seek medical or mental help as control parents. Further community-based studies with comprehensive scrutiny of parental depression and their offspring’s neurodevelopmental conditions would be required to validate our findings. Third, the present study focused on the neurodevelopmental conditions among the offspring of parents with major depressive disorder. Evidence has shown a high comorbidity between major depressive disorder and anxiety disorders. Further investigation would be necessary to determine whether the offspring of parents with anxiety disorders may share a common risk of subsequent neurodevelopmental conditions with those of parents with major depressive disorder and to assess whether there is an additive effect of parental depression and anxiety on the offspring neurodevelopmental conditions.

In conclusion, our study indicated that during a follow-up period, children born to parents with major depressive disorder were more likely to develop ASD, ADHD, tic disorder, and any developmental delay relative to those of parents without major depressive disorder. We further discovered that parental depression was an independent factor affecting the risk of offspring developing neurodevelopmental disorders and various types of developmental delay after adjusting for comorbid perinatal conditions, including being preterm and low birth weight. Our findings indicate that clinicians should closely monitor the developmental conditions of children of parents with major depression. The specific pathomechanisms linking parental depression to offspring neurodevelopment should be clarified through further investigation.

## Data Availability

The NHIRD was released and audited by the Department of Health and Bureau of the NHI Program for the purpose of scientific research (https://www.apre.mohw.gov.tw/). The NHIRD can be accessed through a formal application that is regulated by the Health and Welfare Data Science Center of Ministry of Health and Welfare, Taiwan.
